# Assessing the Quality of Teleconsultations in a Store-And-Forward Telemedicine Network

**DOI:** 10.3389/fpubh.2014.00082

**Published:** 2014-07-16

**Authors:** Richard Wootton, Joanne Liu, Laurent Bonnardot

**Affiliations:** ^1^Norwegian Centre for Integrated Care and Telemedicine, University Hospital of North Norway, Tromsø, Norway; ^2^Faculty of Health Sciences, University of Tromsø, Tromsø, Norway; ^3^Médecins Sans Frontières International, Geneva, Switzerland; ^4^Department of Medical Ethics and Legal Medicine, Paris Descartes University, Paris, France; ^5^Fondation Médecins Sans Frontières, Paris, France

**Keywords:** telemedicine, telehealth, quality assurance, process control, LMICs

## Abstract

Store-and-forward telemedicine in resource-limited settings is becoming a relatively mature activity. However, there are few published reports about quality measurement in telemedicine, except in image-based specialties, and they mainly relate to high- and middle-income countries. In 2010, Médecins Sans Frontières (MSF) began to use a store-and-forward telemedicine network to assist its field staff in obtaining specialist advice. To date, more than 1000 cases have been managed with the support of telemedicine, from a total of 40 different countries. We propose a method for assessing the overall quality of the teleconsultations provided in a store-and-forward telemedicine network. The assessment is performed at regular intervals by a panel of observers, who – independently – respond to a questionnaire relating to a randomly chosen past case. The answers to the questionnaire allow two different dimensions of quality to be assessed: the quality of the process itself and the outcome, defined as the value of the response to three of the four parties concerned, i.e., the patient, the referring doctor, and the organization. It is not practicable to estimate the value to society by this technique. The feasibility of the method was demonstrated by using it in the MSF telemedicine network, where process quality scores, and user-value scores, appeared to be stable over a 9-month trial period. This was confirmed by plotting the cusum of a portmanteau statistic (the sum of the four scores) over the study period. The proposed quality-assessment method appears feasible in practice, and will form one element of a quality assurance program for MSF’s telemedicine network in future. The method is a generally applicable one, which can be used in many forms of medical interaction.

## Introduction

Médecins Sans Frontières (MSF) is a non-governmental humanitarian medical organization that responds to emergency situations and provides medical assistance to those in need. MSF teams provide medical emergency aid in difficult settings around the world, and staff often have to diagnose and treat patients with limited resources (1). In 2010, MSF began to use a store-and-forward telemedicine network to assist its field staff in obtaining specialist advice (2). To date, more than 1000 cases have been managed with the support of telemedicine, from a total of 40 different countries.

In a store-and-forward telemedicine network of this type, doctors in the field refer cases electronically to obtain a second opinion about diagnosis or management. Incoming cases are reviewed by a case coordinator and assigned for reply to one or more appropriate experts. The network therefore operates in a similar way to a bulletin board, with messages being posted by its users. Although formal evidence for the clinical effectiveness of the telemedicine advice obtained through networks of this kind is rather scarce (3, 4), they are known to provide a useful service to referring doctors, and several networks have operated for periods of more than a decade.

### Quality problem

As store-and-forward telemedicine in resource-limited settings is becoming a relatively mature activity, there is a concomitant requirement to implement quality assurance/improvement activities. Indeed, it may be considered unethical not to do so. However, there are few published reports about quality measurement in telemedicine, except in networks concerned with radiology (5), ophthalmology (6), or histopathology (7), many of which are retrospective studies. These reports concern image-based activities, which perhaps lend themselves more readily to quality measurement. The situation in teleconsulting is more complex, being inherently multi-specialty in nature and one where there is often limited knowledge of outcomes. Attempting to measure quality in such a context is more like attempting to measure overall quality in a multi-clinic outpatient department. As far as we are aware, there have been no previous studies of prospective quality measurement in general teleconsulting work in low income countries.

### Objectives

The primary research question was whether a method could be developed for quality measurement in general teleconsulting work in low income countries. The aim of the present work, therefore, was to develop a method for assessing the quality of the teleconsultations being conducted in the MSF telemedicine network, and then to examine its feasibility for routine adoption.

## Materials and Methods

The present study required the development of a method to assess quality and then a demonstration of its feasibility in practice. The work was performed in two stages:

(1) development of a quality-assessment tool
(2) demonstration of feasibility in the MSF telemedicine network

Ethics permission was not required, because patient consent to access the data had been obtained and the work was a retrospective chart review conducted by the organization’s staff in accordance with its research policies.

### Assessment of quality

#### Development of the quality tool

A questionnaire was developed by a consensus between three experienced telemedicine practitioners. It was based on accepted tools used in previous studies (8, 9). The final questionnaire was evaluated and approved by an independent evaluator. The final questionnaire consisted of 17 questions. These concerned the information provided by the referring doctor, the way that the referral was handled in the telemedicine network, the response(s) received from the specialist(s) consulted, and the likely value to the patient, the doctor, and the organization.

#### Definition of quality

We defined quality in terms of two of the three dimensions of the Donabedian model: process and outcome. (The structural dimension is not usually relevant in a telemedicine network of the sort under discussion.) Thus in assessing the quality of a given teleconsultation, there are two principal questions:

(1) was the *process* by which the response was produced satisfactory? i.e., what was the quality of the teleconsultation process itself?
(2) was the *outcome* from the teleconsultation useful? i.e., what was the value of the teleconsultation and to whom?

These questions address separate dimensions of quality, both of concern to network operators. That is, the process for producing a response might be satisfactory, but the response itself could be useless. Or the process could be unsatisfactory, but the response might still be useful.

Both aspects of quality can best be judged by using a panel of assessors. This is because any evaluation will involve subjective judgments, so a panel of observers is more likely to produce an accurate estimate than a single observer. However, it is not feasible to evaluate the quality of every single teleconsultation conducted in the network, so there must be a sampling process by which a case is selected (randomly) for assessment at regular intervals. This leads to a quality-assessment scheme whose main features are summarized in Table 1.

**Table 1:** **Main features of the quality-assessment scheme**.

Sampling of process output	One case per month is selected at random
Panel of assessors	Senior staff (*n* = 12) with experience in the field (mainly doctors)
Evaluation – individual scores	Each panel member responds (independently) to a set of questions, from which the following can be computed: process quality (*q*_p_) and value scores (*v*_p_, *v*_r_, and *v*_o_)
Evaluation – panel scores	Aggregated scores are then calculated to indicate the panel’s overall assessment of process quality (*Q*_p_) and value (*V*_p_, *V*_r_, and *V*_o_), based on the median panel scores
Evaluation – composite score	Finally, a composite score is calculated to reflect the panel’s overall assessment of quality (based on the process quality and value scores)

#### Quality of process

The quality of the teleconsultation process (*q*_p_) can be assessed by the panel members, who can make a judgment about various relevant matters. For example, they can judge whether the referrer provided sufficient information, whether the case was sent promptly to an appropriate expert, whether an answer was obtained sufficiently quickly to be useful and so on. There are 10 questions listed in Table 2 which are relevant to the quality of the process. The scoring system is described in Appendix.

**Table 2:** **Quality-assessment questions**.

Question	Response choices	Quality of the process, *Q*_p_	Value to the patient, *V*_p_	Value to the referring doctor, *V*_r_	Value to the organization, *V*_o_	Value to society, *V*_s_
1. Was the question asked by the referring doctor clear?	Yes/perhaps/no/do not know	X				
2. Did the referrer provide sufficient information?	Yes/perhaps/no/do not know	X				
3. Were any images provided?	Yes/no					
4. If yes, were the images adequate?	Yes/perhaps/no/do not know	X				
5. If no, would some images have helped?	Yes/perhaps/no/do not know	X				
6. Overall, could the referral have been improved?	Yes/perhaps/no/do not know	X				
7. Was the case sent to an appropriate expert?	Yes/perhaps/no/do not know	X		X		
8. Was the answer provided sufficiently quickly?	Yes/perhaps/no/do not know	X		X		
9. Was the answer(s) well-adapted for the local environment?	Yes/perhaps/no/do not know	X		X		
10. Overall, could the answer have been improved?	Yes/perhaps/no/do not know	X		X		
11. Did the telemedicine advice clarify the diagnosis for the doctor and patient?	Yes/perhaps/no/do not know		X	X		
12. Did the suggested action help the doctor manage the patient?	Yes/perhaps/no/do not know		X	X		
13. Do you think that the eventual clinical outcome will be beneficial for the patient?	Yes/perhaps/no/do not know		X		X	
14. Was the consultation useful for the doctors concerned?	Yes/perhaps/no/do not know			X	X	
15. Could the allocation/coordination have been improved?	Yes/perhaps/no/do not know	X				
16. Was the consultation good from the organization’s point of view?	Yes/perhaps/no/do not know				X	X
17. Do you have any comments about this case?	(Free text)					

#### Value of response

The value of the response can be assessed in a similar way by the panel members. There are four domains of interest:

(1) Value to the patient, *v*_p_. After the patient himself, the person best placed to judge this is the referring doctor. It can also be estimated by senior staff in the organization.
(2) Value to the referring doctor, *v*_r_. The person best able to judge this is the referring doctor, but it can also be estimated by senior staff in the organization.
(3) Value to the organization, *v*_o_. This is probably best judged by senior staff in the organization itself.
(4) Value to society as a whole, *v*_s_.

The first three values can be assessed by staff with suitable telemedicine experience. However, assessing the value to society is much more difficult. The value to society of telemedicine will be partly determined by the health care system in the country concerned (mainly, the country where the patient is located), including the degree to which telemedicine has been properly integrated into the chain of health care there. Assessing the value to society as a whole is therefore difficult to do on the basis of a single telemedicine case, and is ignored in what follows. It is worth noting that in a humanitarian context (or a not-for-profit operation), the value to society will be closely aligned with the value to the organization.

Direct measurement of value is not straightforward. In health economics, it is usual to measure the cost-effectiveness of the technique in question and to make a comparison (e.g., with usual practice) to obtain evidence that it does not represent a waste of resources. However, in the context of telemedicine in resource-limited settings, this is not easy to do. First, the costs are distorted, because many staff are volunteers and there may also be donor support, which can be hard to quantify. Second, the clinical effect of telemedicine may be difficult to document, as patients are commonly lost to follow up after their initial encounter.

How else can the “value” of a teleconsultation episode be measured? That is, what is the value to the interested parties? Panel members can form a judgment about whether the telemedicine response clarified the diagnosis, whether the eventual clinical outcome would be beneficial for the patient and so on. There are nine questions listed in Table 2 which are relevant to the value of the response in the domains of interest. The scoring system is described in Appendix.

### Demonstration of feasibility

To demonstrate the feasibility of the proposed approach, a panel of 12 experts was invited to answer the 17 questions about randomly selected telemedicine cases, see Table 2. Cases were chosen at random for a 9-month period. The process was as follows:

(1) the system automatically selected a past case for review at the beginning of each month. The case was chosen randomly from those referred 4–8 weeks previously. If there were fewer than four cases in the period of interest, no case was selected. (The average case submission rate during the period in question was approximately one case per day.)
(2) the members of the quality-assessment panel were notified by email that a case had been chosen for review. The panel comprised mainly senior doctors with previous MSF field experience; there were three other healthcare professionals with telemedicine experience.
(3) panel members logged in to the telemedicine system, viewed the information about the chosen case and answered the questions about the case. The questions had simple, multiple-choice answers, which were presented in a drop-down box for ease of selection. Panel members could not view the answers from any other panel member until they had provided their own.
(4) when at least one set of answers had been provided, the system calculated the quality scores for the case. The four quality scores were values in the range 0–10.

#### Process stability

A control chart was used to examine the stability of the monthly quality scores. Control charts can be plotted for each of the four quality indices, but for simplicity, a grand quality score (GQS) for each case was calculated from the panel’s quality and value scores as

$$G\;=\;Q_{\text{p}}\;+\;V_{\text{p}}\;+\;V_{\text{r}}\;+\;V_{\text{o}}$$

That is, the GQS represents an equi-weighted summation of the four constituent indices. The GQS was transformed to lie in the range 0–10 (0 = worst, 10 = best).

The cusum chart is a well-established and powerful method for identifying changes in a process average. The chart plots the cumulative difference between the recorded values and a target value, which is often chosen to be the process average. The GQS values were plotted as a cusum, using the grand mean as the reference value.

Note that there are two important assumptions underlying the use of control charts: the measurement that is used to monitor the process is distributed according to a normal distribution; it was not necessary to transform the data in the present case. Also, the measurements are assumed to be independent of each other.

## Results

**Figure 1:**
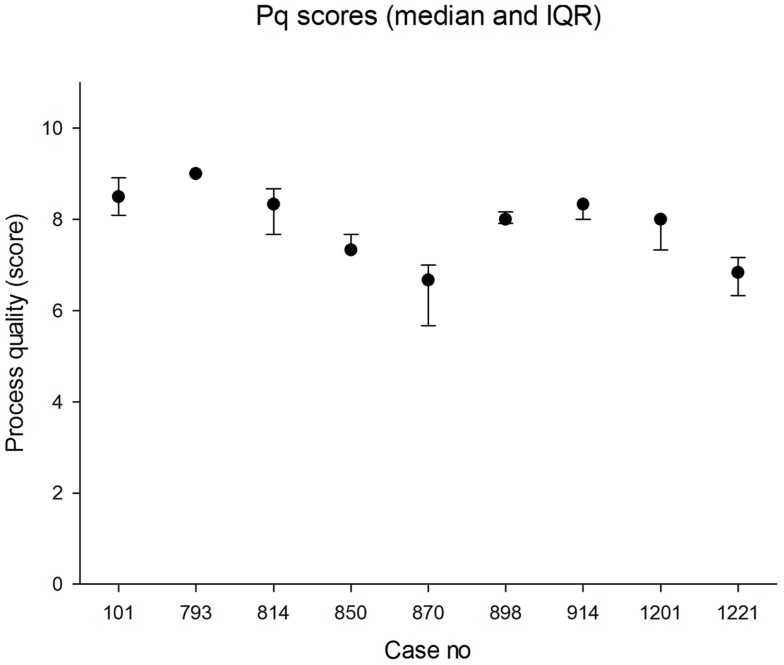
**Median scores for process quality (0 = worst; 10 = best)**. The error bars indicate the 25th and 75th percentiles.

The panel assessed randomly selected cases starting in July 2013. At least four responses were received for each case. The median panel score for process quality was 8.0 (IQR 7.3, 8.7) across the nine cases. The lowest score awarded for process quality by an individual panel member in any case was 4.7 and the highest was 9.0. The median values in each case are shown in Figure 1. There was good agreement between panel members about process quality, i.e., relatively small IQRs for each case.

**Figure 2:**
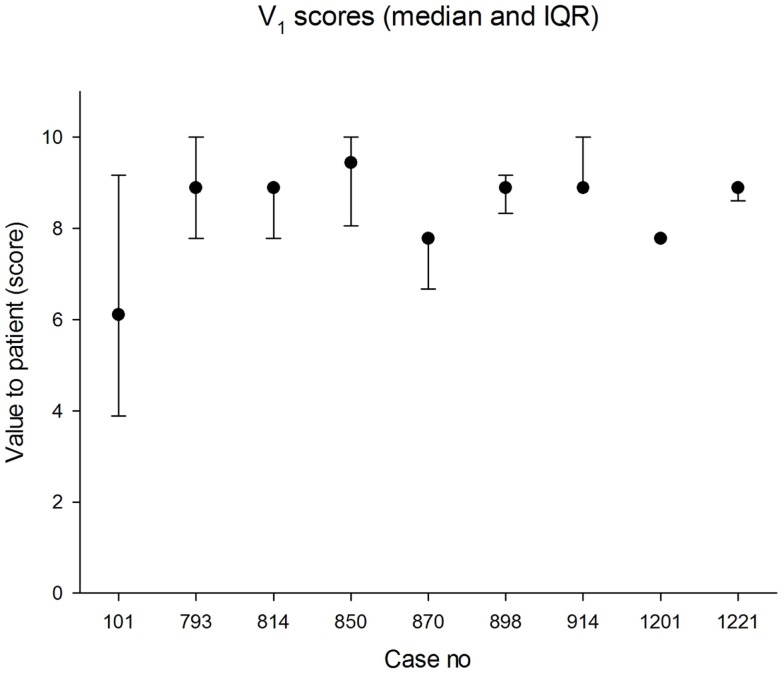
**Median scores for value to patient (0 = worst; 10 = best)**. The error bars indicate the 25th and 75th percentiles.

The median panel score for value to the patient was 8.9 (IQR 7.8, 8.9). The lowest score awarded for value to the patient was 3.3 and the highest was 10. The median values in each case are shown in Figure 2. The agreement between panel members was less good than for process quality.

**Figure 3:**
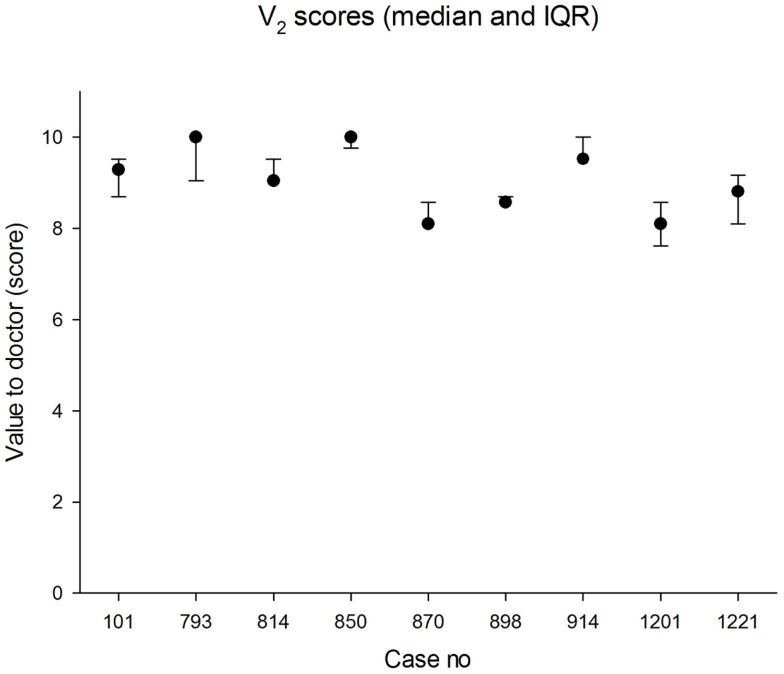
**Median scores for value to referrer (0 = worst; 10 = best)**. The error bars indicate the 25th and 75th percentiles.

The median panel score for value to the doctor was 9.1 (IQR 8.6, 9.5). The lowest score awarded for value to the doctor was 5.7 and the highest was 10.0. The median values in each case are shown in Figure 3. The agreement between panel members was better than for value to the patient.

**Figure 4:**
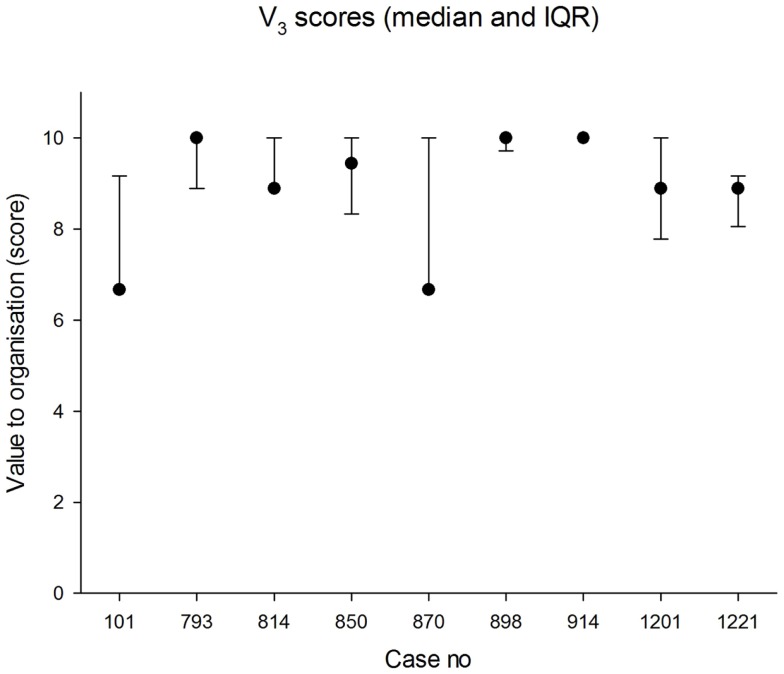
**Median scores for value to the organization (0 = worst; 10 = best)**. The error bars indicate the 25th and 75th percentiles.

The median panel score for value to the organization was 8.9 (IQR 7.2, 10.0). The lowest score awarded for value to the organization was 5.6 and the highest was 10.0. The median values in each case are shown in Figure 4. The agreement between panel members was less good than for value to the doctor.

**Figure 5:**
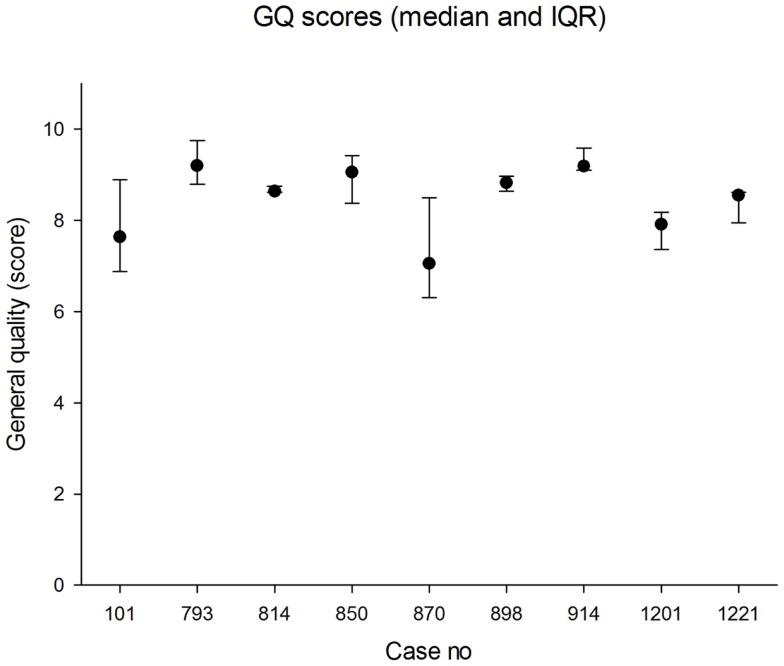
**Median scores for general quality (0 = worst; 10 = best)**. The error bars indicate the 25th and 75th percentiles.

**Figure 6:**
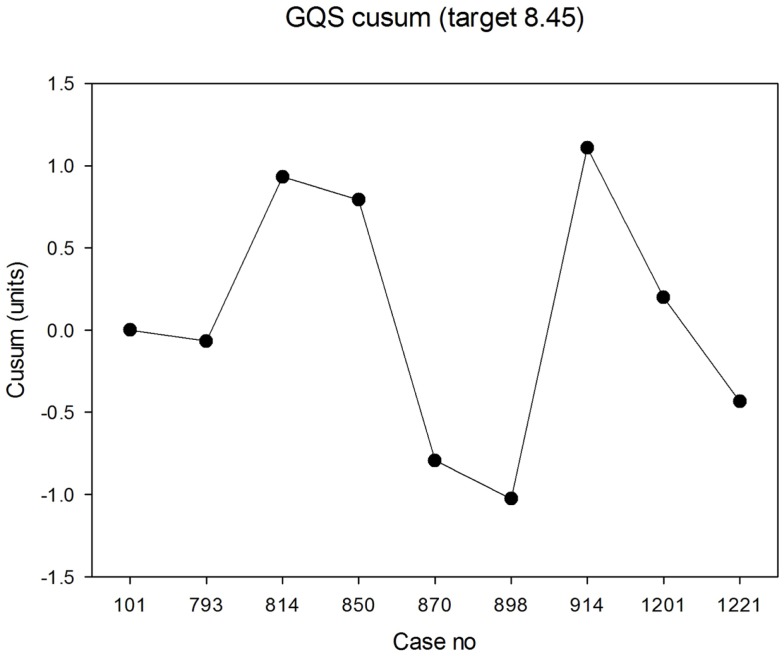
**Cusum of general quality score**.

The median panel GQS was 8.6 (IQR 7.6, 9.2). The lowest individual GQS was 6.1 and the highest was 9.8. The median values in each case are shown in Figure 5. The cusum is shown in Figure 6. There was no evidence that the process was out of control, i.e., with steadily increasing or steadily decreasing values. In fact, over the epoch studied, the cusum was essentially zero at the end, while deviations no larger than ±12% occurred over the study period.

## Discussion

We have developed a quality-assessment scheme for a store-and-forward telemedicine network and demonstrated its feasibility in a real-life clinical setting. There appear to be no previous reports of similar work.

### Relation to other evidence

Previous work on assessment of quality in telemedicine networks has often focused on user satisfaction [e.g., Ref. (10)], which is a related, but different, concept. Most previous quality studies have been retrospective reviews, such as that conducted by Mahnke et al. (11). There have been few attempts to measure the value to the clinician, although Chan et al. investigated this in a real-time teleconsultation network in a high-income country (12).

### Methodological issues

The proposed method was trialed in a real-life telemedicine network, where it was shown to be feasible and appeared to produce useful results. It thus appears suitable for routine adoption. Implicit in the methodology are a number of design decisions.

#### Questionnaire

The size of the questionnaire is likely to influence the number of responses from the panel. The right balance has to be struck between asking too few questions and too many. On one hand, the more questions that are asked, the better the situation can be assessed; but on the other hand, too many questions will discourage the observers from responding, which will make the system less sustainable. In practice, 10–20 questions seems to be a reasonable number.

#### Monitoring and stability

Which index (process quality and the three value domains) is most appropriate for long-term monitoring, in order to measure network performance? Are all four indices of equal importance, or should some be more heavily weighted than others? Should they be monitored collectively, rather than individually? This requires further work.

#### Sampling

How often should cases be sampled and monitoring be performed? On one hand, more frequent sampling will allow closer performance monitoring; on the other hand, it is likely to lead to “observer fatigue.” In practice, we suggest that random sampling of one case per month is about right.

#### Size of panel

How many panel members should give an opinion? The more members there are, the more likely there is to be disagreement between them; on the other hand, the more there are, the better the estimate of the underlying value. In practice, we suggest that 5–10 panel members are about right.

### Quality assurance

Routine measurement of quality on randomly selected cases is only one part of the whole evaluation process and will form one element of an overall quality assurance program for the telemedicine network concerned. Other elements may include obtaining other points of view and follow up reports to assess long-term outcomes concerning the cases and the benefits of the expertise.

### Limitations

The present work has certain limitations. For example, before it could be used routinely, the quality-assessment methodology would require validation. However, it is difficult to validate the proposed indices independently, especially in the context of a telemedicine network operated by a humanitarian organization. Ideally, they should be evidence-based, and of demonstrated validity and reliability (13). Further work is required to find out whether this is possible, since the practical problem of the lack of an obvious gold standard needs to be overcome. Validation may therefore need to rest on psychometric methods (14).

Industrial process control is normally done using an absolute standard as the reference. In the present work, a relative reference value was employed. That is, it represents an assessment of relative quality, which pragmatically, is probably better than no assessment at all. Again, further work is required to find out whether absolute reference standards can be developed.

Finally, the quality of this evaluation relies on the information available for assessing the case. Sampling a case at a particular time may be problematic if there is insufficient feedback on follow up. It also relies on the expertise and experience of the assessor panel. The panel members must be selected carefully and it is important that they have no conflict of interest. This is why it may be better to use independent volunteers, rather than senior staff from the organization running the network.

### Interpretation

The present method provides estimates of the value to the main parties concerned in a teleconsultation, together with an estimate of the quality of the teleconsultation process itself. This is important information for those responsible for the operation of the network. To the best of our knowledge, there has been no information published previously about the quality of general teleconsultations in a store-and-forward network. Yet, if telemedicine is considered sufficiently mature that it can enter routine service, there is an ethical imperative to ensure that it is employed in a cost-effective manner. The method described here provides an instrument for monitoring quality and will form part of the toolset used by the operators of the MSF telemedicine network in future.

Once a method for assessing quality is available, application of industrial process control methodology allows the stability of the network to be monitored. Again, this is important if network operators are to be reassured that quality is not in slow decline. The information may also be valuable in improving the performance of healthcare staff in low-resource settings, which is known to be a difficult problem (15).

The techniques presented in this paper are of wide application. They could potentially be used in non-telemedicine consultations (i.e., conventional, face-to-face consulting), and in industrialized countries as well as resource-limited settings.

## Conclusion

A method for assessing the quality of the teleconsultations in a store-and-forward telemedicine network is proposed. It provides estimates of the quality of the process and the value of the consultation to the main parties involved. A trial of the method showed that it was feasible and that the process in the network studied was stable. The method appears to give useful results. It seems desirable to implement it in other telemedicine projects where it can contribute to the evaluation of practice, something that is necessary in all medical services provided.

## Conflict of Interest Statement

The authors declare that the research was conducted in the absence of any commercial or financial relationships that could be construed as a potential conflict of interest.

## Appendix

### Scoring of questions

The questions set out in Table 2 are presented as multiple choices for simplicity. Scoring for most questions – those in which an affirmative response indicated satisfaction with the item being considered – was No = 1, Perhaps = 2, and Yes = 3. However, some questions required reversed scoring, where an affirmative indicated dissatisfaction: Yes = 1, Perhaps = 2, and No = 3.

Do not know responses were coded as zeroes, i.e., they were treated in the same way as missing values. That is, no distinction was made in the present work between missing values and do not know responses. These may represent cases where assessors experienced particular difficulty in forming a judgment.

#### Individual scores

Scores were first calculated for each individual panel member, as follows. The process quality score from a panel member who had assessed a particular case was calculated as follows:

$$q_{\text{p}}=s_{\text{1}}+s_{\text{2}}+s_{\text{4}}+s_{\text{5}}+s_{\text{6}}+s_{\text{7}}+s_{\text{8}}+s_{\text{9}}+s_{\text{1}0}+s_{\text{15}}$$

where the response elements, *s*_1_, *s*_2_, … refer to the responses in Table 2, reverse-scored where appropriate.

The value scores from a given panel member were calculated as follows:

$$\begin{array}{llll} v_{\text{p}} & =s_{\text{11}}+s_{\text{12}}+s_{\text{13}}\; & & \;\\ v_{\text{r}} & =s_{\text{7}}+s_{\text{8}}+s_{\text{9}}+s_{\text{1}0}+s_{\text{11}}+s_{\text{12}}+s_{\text{14}}\; & & \;\\ v_{\text{o}} & =s_{\text{13}}+s_{\text{14}}+s_{\text{16}}\; & & \; \end{array}$$

In the above calculations, the response elements for a given panel member were aggregated by simple summation. That is, the constituent responses for the questions being used in a particular score were equi-weighted.

For convenience, the values *q*_p_, *v*_p_, *v*_r_, and *v*_o_ were transformed to lie in the range 0–10 (0 = worst, 10 = best).

#### Panel scores

The individual panel member scores were aggregated to produce a panel mean, which represents the best estimate of the underlying true value pertaining to the case in question. In the absence of a compelling reason to use a more complex scheme, the individual scores were equally weighted, i.e., this amounts to placing similar value on the judgment of all members of the panel. For example, the panel’s best estimate of process quality was

$$Q_{\text{p}}=\left(q_{\text{1}}+q_{\text{2}}+\cdot \cdot \cdot +q_{\text{i}}\right)∕n$$

where *Q*_p_ is the panel process quality score, and *q*_1_, *q*_2_ … are the individual scores for process quality from the *n* panel members.
